# TGF-β Induces Surface LAP Expression on Murine CD4 T Cells Independent of Foxp3 Induction

**DOI:** 10.1371/journal.pone.0015523

**Published:** 2010-11-24

**Authors:** Takatoku Oida, Howard L. Weiner

**Affiliations:** Center for Neurologic Diseases, Brigham and Women's Hospital, Harvard Medical School, Boston, Massachusetts, United States of America; New York University, United States of America

## Abstract

**Background:**

It has been reported that human FOXP3^+^ CD4 Tregs express GARP-anchored surface latency-associated peptide (LAP) after activation, based on the use of an anti-human LAP mAb. Murine CD4 Foxp3^+^ Tregs have also been reported to express surface LAP, but these studies have been hampered by the lack of suitable anti-mouse LAP mAbs.

**Methodology/Principal Findings:**

We generated anti-mouse LAP mAbs by immunizing TGF-β^−/−^ animals with a mouse *Tgfb1*-transduced P3U1 cell line. Using these antibodies, we demonstrated that murine Foxp3^+^ CD4 Tregs express LAP on their surface. In addition, retroviral transduction of Foxp3 into mouse CD4^+^CD25^−^ T cells induced surface LAP expression. We then examined surface LAP expression after treating CD4^+^CD25^−^ T cells with TGF-β and found that TGF-β induced surface LAP not only on T cells that became Foxp3^+^ but also on T cells that remained Foxp3^−^ after TGF-β treatment. GARP expression correlated with the surface LAP expression, suggesting that surface LAP is GARP-anchored also in murine T cells.

**Conclusions/Significance:**

Unlike human CD4 T cells, surface LAP expression on mouse CD4 T cells is controlled by Foxp3 and TGF-β. Our newly described anti-mouse LAP mAbs will provide a useful tool for the investigation and functional analysis of T cells that express LAP on their surface.

## Introduction

TGF-β controls immune responses by multiple mechanisms including the suppression of Th1 and cytotoxic lymphocytes, and the induction of Th17 cells depending on the context [1]. TGF-β is first synthesized as pro-TGF-β and is then intracellularly processed by furin proprotein convertase to form a latent TGF-β complex which consists of non-covalently associated dimmers of the N-terminal region of pro-TGF-β (latency-associated peptide, LAP) and of the C-terminal region of pro-TGF-β (mature TGF-β) [2]. Expression of pro-TGF-β, LAP, latent TGF-β and/or mature TGF-β (hereafter referred as LAP/TGF-β) on mouse CD4 T cells was first reported in 2001 by Nakamura et al. [3]. They proposed that CD4^+^CD25^+^ regulatory T cells (Tregs) mediated their suppressive function by presenting active TGF-β to effector cells in a cell-cell contact manner. They used a polyclonal chicken anti-TGF-β antibody and a monoclonal anti-human LAP mAb (clone 27232) for FACS staining of mouse CD4 T cells. Our laboratory has also reported the presence of surface LAP^+^ on mouse T cells using a polyclonal goat anti-human LAP antibody [4], [5]. However, use of a polyclonal antibody is problematical due to the inherent variance between different polyclonal preparations. The anti-LAP mAb (clone 27232) used by Nakamura, et al., was raised against recombinant human LAP (R&D Systems). Although Nakamura et al. used this antibody to stain mouse CD4 T cells [3], in our hands, we did not find that this anti-human LAP mAb cross-reacted with mouse LAP. Thus, although clone 27232 stained human *TGFB1*-transduced cells [6], it did not stain mouse *Tgfb1*-tranduced cells at all (Figure S1). To overcome these problems, a fully characterized anti-mouse LAP mAb would be required for staining mouse T cells.

Recently, by using the anti-human LAP mAb 27232 [7], [8], it was reported that human FOXP3^+^ Tregs express surface LAP after activation and that the surface LAP is anchored by GARP/LRRC32 [8], [9].

We raised anti-mouse LAP mAbs by immunizing TGF-β^−/−^ mice with mouse *Tgfb1*-transduced cells, and used them to stain mouse CD4 T cells. We found that the majority of mouse Foxp3^+^ CD4 T cells expressed surface LAP after activation. Surface LAP was induced by *Foxp3*-transduction into mouse CD4^+^CD25^−^ T cells and by addition of TGF-β to mouse CD4^+^CD25^-^ T cell cultures. In contrast to human T cells [8], TGF-β induced surface LAP not only on T cells that converted to Foxp3^+^ but also on T cells in which Foxp3 was not expressed. GARP expression correlated with the surface LAP expression suggesting that surface LAP is anchored by GARP.

## Results

### Generation of anti-mouse LAP mAbs

We used mouse *Tgfb1*-transduced P3U1 (P3U1-muTGF-β cells) cells as an immunogen. We have recently shown that human *TGFB1*-transduced P3U1 (P3U1-huTGF-β) cells express LAP/TGF-β on the surface [6]. Surface expression of murine LAP was also expected on P3U1-muTGF-β cells since we found that anti-TGF-β (clone 9016) surface stained P3U1-muTGF-β cells as well as P3U1-huTGF-β cells (Figure S1). We elected to immunize TGF-β-deficient mice. TGF-β^−/−^ mice manifest an autoimmune syndrome and die at 3–4 wks after birth [10], [11]. We attempted to prolong their life by injecting galectin-1, which has been reported to suppress other autoimmune diseases [12], starting at day 7 of birth. P3U1-muTGF-β cells were injected i.p. every other day 5 times beginning at day 8 after birth, and spleen cells were taken at day 22 after birth and fused with P3U1 myeloma cells. The hybridoma cells were grown in hypoxanthine-aminopterin-thymidine (HAT)-supplemented methylcellulose medium. Approximately 2,800 clones were picked from the plates, and transferred to hypoxanthine-thymidine (HT)-supplemented DMEM in 96-well plates. The culture supernatants were screened by surface staining of P3U1-muTGF-β cells by FACS. Thirty-six positive clones were selected and recovered (TW7 series) (Figure S2). Of the 36 clones, 32 clones were IgG and 4 clones were IgM. To check their specificity, we tested the ability of the antibodies to immunoprecipitate Flag-tagged mouse LAP (Flag-mLAP) produced by retrovirally Flag-mLAP-transduced P3U1 cells. Of the 32 IgG clones, 26 clones, including TW7-16B4 and TW7-20B9, immunoprecipitated Flag-mLAP (Figure S3, underlined) and thus were true anti-mouse LAP mAbs. Several clones, including TW7-28G11, did not immunoprecipitate Flag-mLAP (Figure S3). TW7-28G11, however, stained human latent TGF-β-coated beads, but not human LAP- or human active TGF-β-coated beads (Figure S4A). TW7-28G11 immunoprecipiated Flag-mLAP only when active TGF-β was exogenously added to Flag-mLAP solution (Figure S4B), and immunoprecipiated pro-TGF-β and latent TGF-β from the culture supernatant of P3U1-muTGF-β cells (Figure S5A). These results indicate that TW7-28G11 is a conformation specific anti-mouse/human latent TGF-β/pro-TGF-β mAb which recognize LAP and TGF-β in combination. The specificity of some clones, including TW7-16B4, TW7-20B9 and TW7-28G11, were further confirmed by testing their ability to detect mouse pro-TGF-β and/or LAP by Western blot (Figure S5B), and by their ability to immunoprecipiate pro-TGF-β and latent TGF-β from culture supernatant of P3U1-muTGF-β cells (Figure S5A).

### Surface LAP expression on mouse Foxp3^+^ CD4 T cells

**Figure 1:**
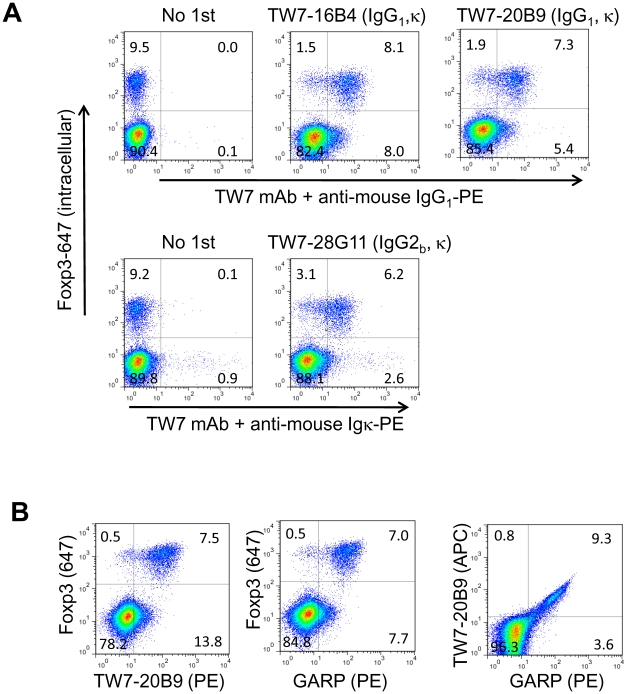
Surface LAP/TGF-β expression on mouse activated CD4 T cells. (A) BALB/c CD4 T cells were stimulated with plate-bound anti-CD3/anti-CD28 for 2 days and rested for 1 day. The cells were surface stained with anti-LAP mAbs using PE-labeled secondary antibodies, then intracellularly stained with anti-Foxp3-Alexa Fluor647. Staining with representative clones, anti-LAP mAbs TW7-16B4 and TW7-20B9, and anti-latent TGF-β/pro-TGF-β mAb TW7-28G11, are shown. (B) Activated BALB/c CD4 T cells were stained with anti-LAP TW7-20B9 (surface) and anti-Foxp3 (intracellular) (left), with anti-GARP (surface) and anti-Foxp3 (intracellular), or anti-LAP TW7-20B9 (surface) and GARP (surface) (right).

It has been reported that human FOXP3 Tregs express surface LAP after activation [7], [8] by a GARP-mediated anchoring mechanism [8], [9]. We tested our anti-LAP/TGF-β mAbs for their ability to stain pre-activated mouse CD4 T cells. CD4 T cells were stimulated with plate-bound anti-CD3/anti-CD28 for 2 days and rested for 1 day. Following this, they were surface stained with anti-LAP/TGF-β mAbs using PE-labeled anti-mouse IgG_1_ (for IgG_1_ subtype clones) or anti-mouse Igκ secondary antibody (for non-IgG_1_ clones), then fixed and intracellularly stained with anti-Foxp3-Alexa Fluor647. Of the 36 potential anti-LAP/TGF-β candidate clones, 31 clones surface stained Foxp3^+^ cells. Three representative clones (TW7-16B4, TW7-20B9, and TW7-28G11) are shown in Figure 1A and all 36 clones are shown in Figure S6. It should be noted that 24 of the 26 clones which immunoprecipitated Flag-mLAP as described above stained Foxp3^+^ CD4 T cells with a similar pattern. Among them, TW7-16B4 produced the highest staining signal followed by TW7-20B9. An anti-pro-TGF-β/latent TGF-β clone, TW7-28G11, also stained Foxp3^+^ CD4 T cells (Figure 1A and Figure S6), suggesting that surface LAP exists as pro-TGF-β and/or latent TGF-β rather than free LAP without mature TGF-β. Surface LAP staining strongly correlated with GARP expression (Figure 1B), indicating that surface LAP on mouse Foxp3^+^ CD4 Tregs is also anchored by GARP as on human FOXP3^+^ CD4 Tregs.

**Figure 2:**
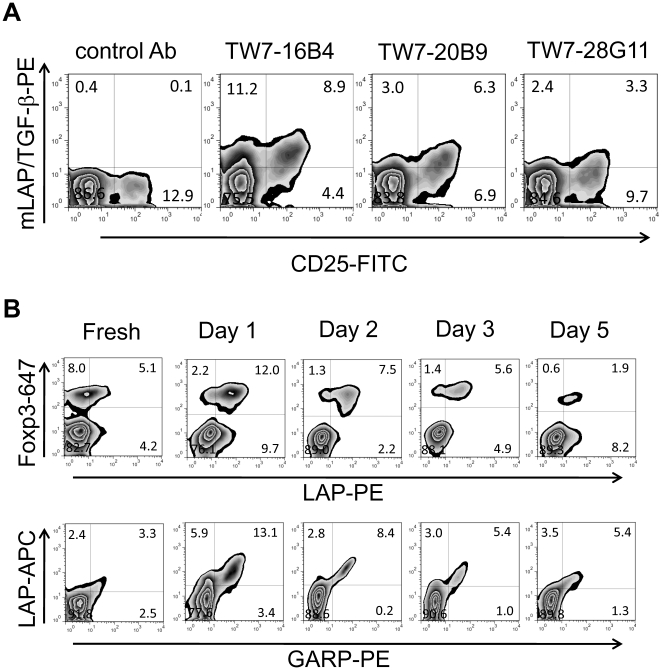
Surface LAP/TGF-β expression on mouse unstimulated CD4 T cells and time course analysis. (**A**) Freshly prepared BALB/c CD4 T cells were surface stained with PE-conjugated anti-LAP/TGF-β mAbs (TW7-16B4, TW7-20B9, or TW7-28G11), anti-CD25-FITC, anti-CD4-APC, and 7-AAD. CD4^+^7-AAD^−^ cells were gated. (B) BALB/c CD4 T cells were stimulated with plate-bound anti-CD3/anti-CD28 for two days, and then split in 10% FBS-IMDM containing 100 U/ml IL-2. The cells were surface stained with PE-conjugated anti-LAP TW7-20B9 followed by anti-Foxp3-Alexa Fluor647 (intracellular staining) (upper panels), or with APC-conjugated anti-LAP TW7-20B9 and GARP-PE (lower panels).

For further analysis we selected TW7-16B4 (IgG_1_, κ) and TW7-20B9 (IgG_1_, κ) as the highest staining anti-LAP clones, and TW7-28G11 (IgG_2b_, κ) as an anti-pro-TGF-β/latent TGF-β clone. These clones were used with secondary antibodies or as antibodies directly labeled with PE or Allophycocyanin (APC). We tested whether unstimulated CD4 T cells also express surface LAP using the direct conjugates. We found that freshly prepared mouse CD4^+^25^+^ T cells also weakly expressed surface LAP (Figure 2A). We also investigated the time course of surface LAP expression. We found that surface LAP expression on Foxp3^+^ cells peaked on days 1 and 2, and then gradually decreased when the cells were rested (days 3 and 5) (Figure 2B, upper panels). We found that GARP was co-expressed with LAP in all time points (Figure 2B, lower panels).

### Foxp3-induced surface LAP expression

**Figure 3:**
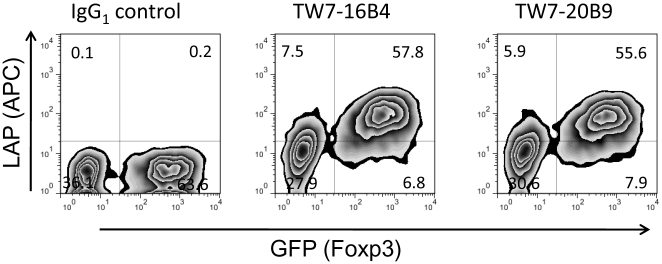
Induction of surface LAP by Foxp3 transduction. BALB/c CD4^+^CD25^−^ T cells were stimulated with plate-bound anti-CD3/anti-CD28 and retrovirally transduced with pMCs-Foxp3-IRES-GFP vector. The cells were re-stimulated with plate-bound anti-CD3/anti-CD28 for 14 hrs and transferred to uncoated wells. 2 days after re-stimulation, the cells were stained with anti-LAP TW7-16B4 or TW7-20B9 using anti-mouse IgG_1_-APC secondary antibody.

We then asked whether surface LAP expression is controlled by Foxp3. We found that retroviral Foxp3 transduction into mouse CD4^+^CD25^−^ T cells induced surface LAP (GFP^+^ population vs. GFP^−^ population in Figure 3). This result demonstrates that surface LAP is under control of Foxp3.

### TGF-β-induced surface LAP expression

**Figure 4:**
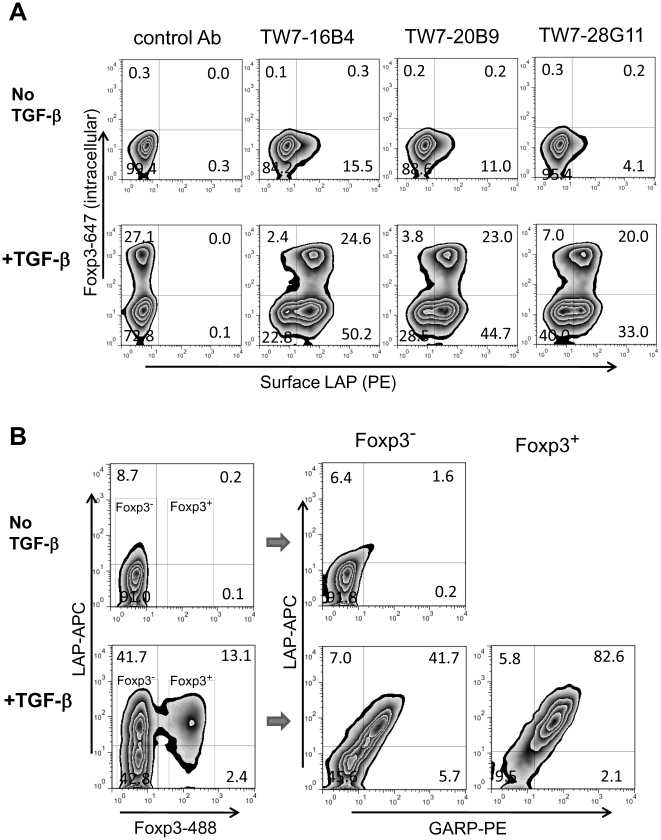
Induction of surface LAP by TGF-β. (**A**) BALB/c CD4^+^CD25^−^ T cells were stimulated with plate-bound anti-CD3/anti-CD28 without (upper panels) or with 10 ng/ml recombinant TGF-β (lower panels) for 2 days and rested for 2 days. The cells were surface stained with anti-LAP TW7-16B4 or TW7-20B9, or anti-latent TGF-β/pro-TGF-β TW7-28G11 using goat anti-mouse Ig-PE secondary antibody, then fixed, and intracellularly stained with anti-Foxp3-Alexa Fluor647. (B) BALB/c CD4^+^CD25^−^ T cells were stimulated with/without TGF-β, and then surface stained with ACP-conjugated anti-LAP TW7-20B9 and GARP-PE, followed by intracellular staining with anti-Foxp3-Alexa Fluor488. Foxp3^−^ and Foxp3^+^ cells populations were gated and plotted by LAP and GARP expression.

TGF-β converts Foxp3^−^ CD4 T cells into induced Foxp3^+^ Tregs (iTregs) [1]. To determine whether iTregs also express surface LAP, we stimulated mouse CD4^+^CD25^−^ T cells in the presence or absence of recombinant TGF-β and checked for surface LAP expression. As expected ∼25% of CD4^+^CD25^-^ T cells were converted to Foxp3^+^ iTregs in presence of TGF-β (Figure 4A). We found that these iTregs expressed surface LAP. Interestingly, the Foxp3^−^-remaining cells also became surface LAP^+^ cells following culture in the presence of TGF-β. GARP expression correlated with surface LAP expression on both Foxp3^+^ cells and Foxp3^−^ cells (Figure 4B), suggesting that surface LAP is GARP-dependent not only on natural Tregs and iTregs cells but also on non-Tregs.

It is possible that surface LAP expression on natural Foxp3^+^ Tregs might also be maintained by TGF-β produced by Tregs themselves. We found, however, that the ALK5 inhibitor or anti-TGF-β 1D11 did not affect surface LAP expression or GARP expression on Foxp3^+^ Tregs (Figures S7 and S8). Thus, these results suggest that surface LAP expression on Foxp3^+^ Tregs is independent of TGF-β.

## Discussion

The existence and function of surface LAP on Tregs has been a matter of debate. Contrary to the first report by Nakamura et al. [3], Shevach's group questioned the function of TGF-β in Treg-mediated suppression [13], and their staining of mouse T cells was quite faint, if at all present [14]. As a part of our investigation of TGF-β, we found that the anti-human LAP mAb 27232 used by Nakamura et al. does not cross-react with mouse LAP (Figure S1). In this report, we raised anti-mouse LAP mAbs by immunization of TGF-β^−/−^ mice and revisited the existence of surface LAP on mouse CD4 T cells. We found that anti-mouse LAP mAbs stained majority of Foxp3^+^ Tregs, but not Foxp3^−^ T cells after activation (Figure 1A). Fresh CD4^+^CD25^+^ T cells also expressed surface LAP at a weak level (Figure 2A). Thus our results establish that mouse Foxp3^+^ Tregs do express surface LAP. It should be mentioned, however, that it is not yet determined whether surface LAP/TGF-β has a functional contribution to Treg-mediated suppression.

Using the anti-LAP mAb 27232 [7], [8] it was recently reported that human FOXP3^+^ Tregs express surface LAP and that the surface LAP is anchored by GARP [8], [9]. It appears that this is also the case with mouse CD4 T cells since GARP expression strongly correlated with surface LAP expression (Figure 1B and Figure 2B). We recently reported the occurrence of GARP-independent, GRP78-associated surface LAP on *TGFB1*-transduced cells [6]. It is unknown at this time whether GARP-independent surface LAP also can be seen on T cells.

In humans, TGF-β-induced FOXP3^+^ CD4 T cells do not express surface LAP or GARP [8]. On the contrary, in mice, not only did TGF-β-induced Foxp3^+^ CD4 T cells express surface LAP and GARP, but TGF-β-exposed CD4^+^CD25^−^ T cells that did not become Foxp3^+^ CD4 T cells also expressed surface LAP and GARP (Figure 4B). Some Foxp3^−^ CD4 T cells also expressed surface LAP/TGF-β without exogenous of TGF-β. We do not know whether this LAP/TGF-β expression was induced by TGF-β in an autocrine fashion or occurred independent of TGF-β. However, TGF-β signaling seems not absolutely required for surface LAP expression since natural Foxp3^+^ Tregs maintained surface LAP expression even when TGF-β signaling was blocked (Figure S7). Thus, surface LAP expression may be controlled independently by Foxp3 and TGF-β signaling.

In summary, we raised anti-mouse LAP mAbs and revisited surface LAP expression on mouse CD4 T cells. We found that Foxp3^+^ Tregs expressed surface LAP and that surface LAP is induced by forced expression of Foxp3 or by TGF-β irrespective of Foxp3 induction. Furthermore, surface LAP expression strongly correlated with GARP, suggesting that surface LAP is GARP-mediated. These newly described anti-mouse LAP mAbs will provide a useful tool for functional analysis of T cells that express LAP on their surface.

## Materials and Methods

### Generation of anti-mouse LAP mAbs (TW7 series)

Mice were housed in a pathogen-free environment and the animal protocols were approved according to the guidelines of the Committee on Animals of Harvard Medical School (Protocol No. 02683). TGF-β^−/−^ mice [10] were injected i.p. with 20 µg galectin-1 (Sigma-Aldrich) [12] every other day starting at 7 day after birth to prevent the fatal autoimmunity seen in TGF-β^−/−^ mice [11]. Mouse *Tgfb1*-transduced P3U1 (P3U1-muTGF-β) cells (clone #11) were injected i.p. at 1-4×10^6^ cells (in 10–25 µl PBS) every other day 5 times starting at 8 days after birth. At age 22 days, the spleen cells were fused with P3U1 myeloma cells, and the hybridoma cells were plated in methylcellulose medium (ClonaCell-HY, Stemcell Technologies). Screening was conducted by surface staining of P3U1-muTGF-β cells by FACS. Anti-mouse LAP specificity was confirmed by immunoprecipitation of recombinant Flag-tagged mouse LAP (lacking C-terminal mature TGF-β sequence) (Flag-mLAP) [15], immunoprecipitation of pro-TGF-β and latent TGF-β, staining recombinant human latent TGF-β (R&D Systems)-coated polystyrene beads, and/or staining recombinant human TGF-β (R&D Systems) coated polystyrene beads.

### Other antibodies and reagents

Anti-human LAP mAb clone 27232, anti-TGF-β mAb clone 9016, and biotinylated goat anti-LAP (BAF246) were obtained from R&D Systems. Anti-mouse CD3 (145-2C11), anti-mouse CD28 (37.51), Allophycocyanin (APC)-labeled goat anti-mouse Ig, PE- or APC-labeled anti-mouse IgG_1_ (A85-1), and PE-labeled anti-mouse Igκ (187.1) were from BD Biosciences. PE-labeled anti-mouse GARP (YGIC86), and Alexa Fluor647-labeled anti-Foxp3 (FJK-16s) were from eBioscience. Alexa Fluor488-labeled anti-Foxp3 (150D) was from Biolegend. TGF-β receptor I kinase inhibitor (ALK5 inhibitor II) was from EMD/Calbiochem. Anti-Flag mAb (M2) was from Sigma-Aldrich. (caga)_12_-MLP-Luc TGF-β reporter plasmid [16], [17] was kindly provided by Dr. D. Vivien (the Universite' de Caen, Daix, France). Mv1Lu cells (ATCC) were stably transfected with (caga)_12_-MLP-Luc plasmid and used for testing dose-response of ALK5 inhibitor II in TGF-β bioassay.

### CD4 T cell stimulation and FACS staining

CD4 T cells were separated from BALB/c mice (The Jackson Laboratories) or C57BL/6 background Foxp3-GFP knock-in (Foxp3-KI) mice [18] using MACS CD4 purification kit (Miltenyi Biotec). When CD4^+^CD25^−^ T cells were prepared, biotinylated anti-CD25 antibody was additionally mixed to the MCAS antibody cocktail. T cells were stimulated with plate-bound anti-CD3 and anti-CD28 for 2 days. The cells were split into non-coated wells and rested for 1 day, then stained by FACS. Surface LAP staining was conducted by either PE- or APC-directly conjugated anti-mouse LAP mAbs, or unconjugated anti-mouse LAP mAbs followed by PE- or APC-conjugated goat anti-mouse Ig, monoclonal anti-mouse IgG_1_ or monoclonal anti-mouse Igκ secondary antibody. Intracellular Foxp3 staining was done with Alexa Fluor647- or Alexa Fluor488-labeled anti-Foxp3 using Foxp3 Staining Buffer Set (eBioscience).

### Retroviral transduction

Retroviral vector pMCs-IRES-GFP [19], ecotropic retroviral packaging cell line Plat-E [20] were kindly provided by Dr. Kitamura (Tokyo Univ., Tokyo, Japan). Foxp3 was cloned into pMCs-IRES-GFP vector, and the retroviral supernatant was produced by Plat-E. Mouse CD4^+^25^−^ T cells from BALB/c mice pre-activated with plate-bound anti-CD3 and anti-CD28 for 30 hrs were infected with Foxp3 ecotropic retrovirus by centrifugation at 3,000 rpm for 1 hr. 1 day after infection, the cells were split onto a non-coated wells, and rested. The transduced cells were re-stimulated with plate-bound anti-CD3/anti-CD28 for 14 hrs, rested for 2 days, and surface stained with anti-LAP mAbs and then intracellularly with anti-Foxp3.

## Supporting Information

Figure S1 **Negative staining of mouse TGF-β-transduced cells with anti-human LAP mAb 27232.** Non-transduced P3U1 cells (green), human TGF-β gene (*TGFB1*)-transduced P3U1 cells (clone #32) (blue), or mouse TGF-β gene (*Tgfb1*)-transduced P3U1 (clone #11) cells (red) were surface stained with anti-TGF-β mAb 9016 (left) or with anti-human LAP mAb 27232 (right). Note that mouse TGF-β-transduced P3U1 cells were later found positive with anti-mouse LAP mAbs as shown in Figure S2.
(PDF)

Figure S2 **Staining of mouse TGF-β-transduced P3U1 cells with TW7 anti-LAP/TGF-β** **candidate clones.** Mouse TGF-β-transduced P3U1 (clone #11) cells (GFP^+^) mixed with non-transduced P3U1 cells (GFP(^-^)) were surface stained with culture supernatants of anti-LAP/TGF-β candidate clones (TW7 series) using goat anti-mouse Ig-APC after Fc receptor blocking. Immunoglobulin subtypes are also shown in the figures. Clones identified as anti-LAP in Fig. 3 are underlined.
(PDF)

Figure S3 **Immunoprecipitation of Flag-tagged mouse LAP with TW7 anti-LAP/TGF-β** **candidate clones.** Culture supernatant of P3U1 cells transduced with retroviral pMCs vector carrying Flag-tagged mouse LAP lacking TGF-β sequence (Flag-mLAP) was immunoprecipitated with anti-LAP/TGF-β candidate clones using anti-mosue IgG BioMag Plus (Polysciences). The immunoprecipitated samples were run on SDS-PAGE under reducing conditions and blotted with anti-Flag mAb M2. Ig H chain and Ig L chain were detected at 55 kDa and at 25 kDa, respectively, and Flag-mLAP migrated at 43 kDa. Clones that immunoprecipitaed Flag-mLAP were marked under the clone numbers. C, MOPC21 IgG_1_ control; 1, TW7-1C12 (IgG_1_); 2, TW7-3G11 (IgM); 3, TW7-4G7 (IgG_1_); 4, TW7-5A1 (IgG_1_); 5, TW7-5B2 (IgG_1_); 6, TW7-5B5 (IgG_1_); 7, TW7-5D4 (IgG_1_); 8, TW7-5F5 (IgG_1_); 9, TW7-5G10 (IgG_1_); 10, TW7-6B3 (IgG_1_); 11, TW7-7C7 (IgG_1_); 12, TW7-7G7 (IgG_1_); 13, TW7-7H4 (IgG_1_); 14, TW7-8C11 (IgG_1_); 15, TW7-10C10 (IgG_1_); 16, TW7-11G5 (IgG_1_); 17, TW7-12E2 (IgG_1_); 18, TW7-13C5 (IgG_1_); 19, TW7-13C8 (IgG_1_); 20, TW7-13D7 (IgG_1_); 21, TW7-13E12 (IgG_1_); 22, TW7-16A2 (IgG_1_); 23, TW7-16B4 (IgG_1_); 24, TW7-17G8 (IgM); 25, TW7-18C4 (IgG_2a_ or _2b_); 26, TW7-18C9 (IgG_2a_ or _2b_); 27, TW7-20B9 (IgG_1_); 28, TW7-22F7 (IgG_1_); 29, TW7-22F9 (IgG2a or 2b); 30, TW7-22H5 (IgG_1_); 31, TW7-23D12 (IgG_1_); 32, TW7-24B11 (IgG_1_); 33, TW7-24E3 (IgM); 34, TW7-24G5 (IgG_1_); 35, TW7-26E10 (IgM); 36, TW7-28G11 (IgG_2b_).
(PDF)

Figure S4 **Characterization of TW7-28G11 clone.** (A) Recombinant human LAP- (left), human latent TGF-β- (middle), or human active TGF-β- (right) coated polystyrene beads were stained with TW7-28G11 mAb using goat anti-mouse Ig-APC. (B) Culture supernatant of Flag-mLAP-transduced P3U1 cells with/without exogenously added recombinant human TGF-β was immunoprecipitated with TW7-28G11 or control Ab. The samples were run on SDS-PAGE under reducing conditions and blotted with anti-Flag M2 antibody.
(PDF)

Figure S5 **Western blotting and immunoprecipitation of LAP/TGF-β by TW7 mAbs.** (A) Culture supernatant of P3U1-muTGF-β (clone #11) cells (lane 1), or immunoprecipitated samples from P3U1-muTGF-β culture supernatant with TW7-7H4 (lane 2), TW7-16B4 (lane 3), TW7-20B9 (lane 4), TW7-22F7 (lane 5), TW7-28G11 (lane 6), or or IgG_1_ control MOPC21 (lane 7) were run on SDS-PAGE under non-reducing conditions, and blotted with biotinylated goat anti-LAP Ab. (B) Culture supernatant of P3U1-muTGF-β (clone #11) cells were run on SDS-PAGE under non-reducing conditions and blotted with TW7-16B4 (lane 1), TW7-20B9 (lane 2), TW7-28G11 (lane 3), or biotinylated goat anti-LAP (lane 4).
(PDF)

Figure S6 **Staining of pre-activated mouse CD4 T cells with TW7 anti-LAP/TGF-β** **mAb series.** BALB/c CD4 T cells were stimulated with plate-bound anti-CD3/anti-CD28 for 2 days and rested 1 day. The cells were surface stained with TW7 anti-LAP/TGF-β mAbs using PE-labeled anti-mouse IgG_1_ or anti-mouse Igκ secondary antibodies, then intracellularly stained with anti-Foxp3-Alexa Fluor647 as Figure 2A. Staining with all 36 TW7 clones was shown.
(PDF)

Figure S7 **Surface LAP expression under TGF-β blocking conditions.** B6 background Foxp3-GFP knock-in CD4 T cells were stimulated with plate-bound anti-CD3/anti-CD28 in presence of 10 ng/ml recombinant human TGF-β, 1 µM ALK5 inhibitor II (Figure S8), or 50 µg/ml anti-TGF-β mAb 1D11 for 2 days, and rested for 1 day. The cells were stained with anti-LAP TW7-16B4 using anti-mouse IgG_1_-APC secondary antibody and anti-GARP-PE. The quadrants were set by isotype control staining
(PDF)

Figure S8 **Dose response curve of ALK5 inhibitor II.** (A) Mv1Lu cells stably transfected with (caga)_12_-MLP-Luc vector were cultured in the presence of recombinant human TGF-β for 8 hrs, and luciferase was measured. (B) Mv1Lu-(caga)_12_-MLP-Luc cells were cultured in presence of 100 pg/ml recombinant human TGF-β with various concentrations of ALK5 inhibitor II for 8 hrs, and luciferease was measured.
(PDF)
