# Supplementary figures and images for: TGF-β Induces Surface LAP Expression on Murine CD4 T Cells Independent of Foxp3 Induction
Source: PLoS One. 2010 Nov 24;5(11):e15523. doi: 10.1371/journal.pone.0015523 (PMC2991360; doi:10.1371/journal.pone.0015523)

anti-TGF- $\beta$  clone 9016

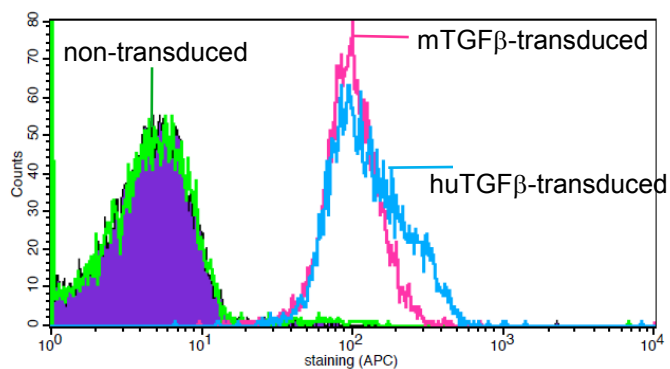

anti-human LAP clone 27232

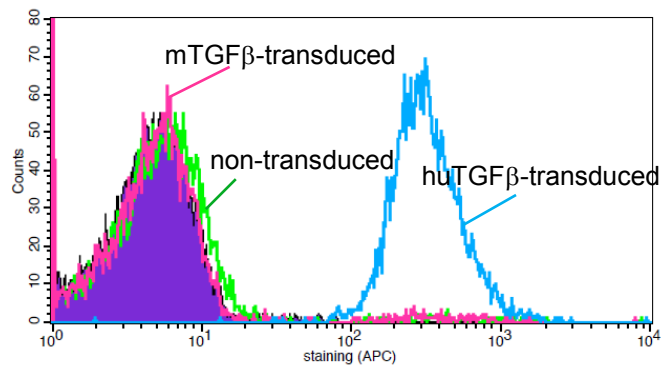

Figure S1

Supplement: Figure S1 — Negative staining of mouse TGF-β-transduced cells with anti-human LAP mAb 27232. Non-transduced P3U1 cells (green), human TGF-β gene (TGFB1)-transduced P3U1 cells (clone #32) (blue), or mouse TGF-β gene (Tgfb1)-transduced P3U1 (clone #11) cells (red) were surface stained with anti-TGF-β mAb 9016 (left) or with anti-human LAP mAb 27232 (right). Note that mouse TGF-β-transduced P3U1 cells were later found positive with anti-mouse LAP mAbs as shown in Figure S2. (PDF) [file pone.0015523.s001.pdf]

TW7 sup +  $\alpha$ Mouse Ig-APC

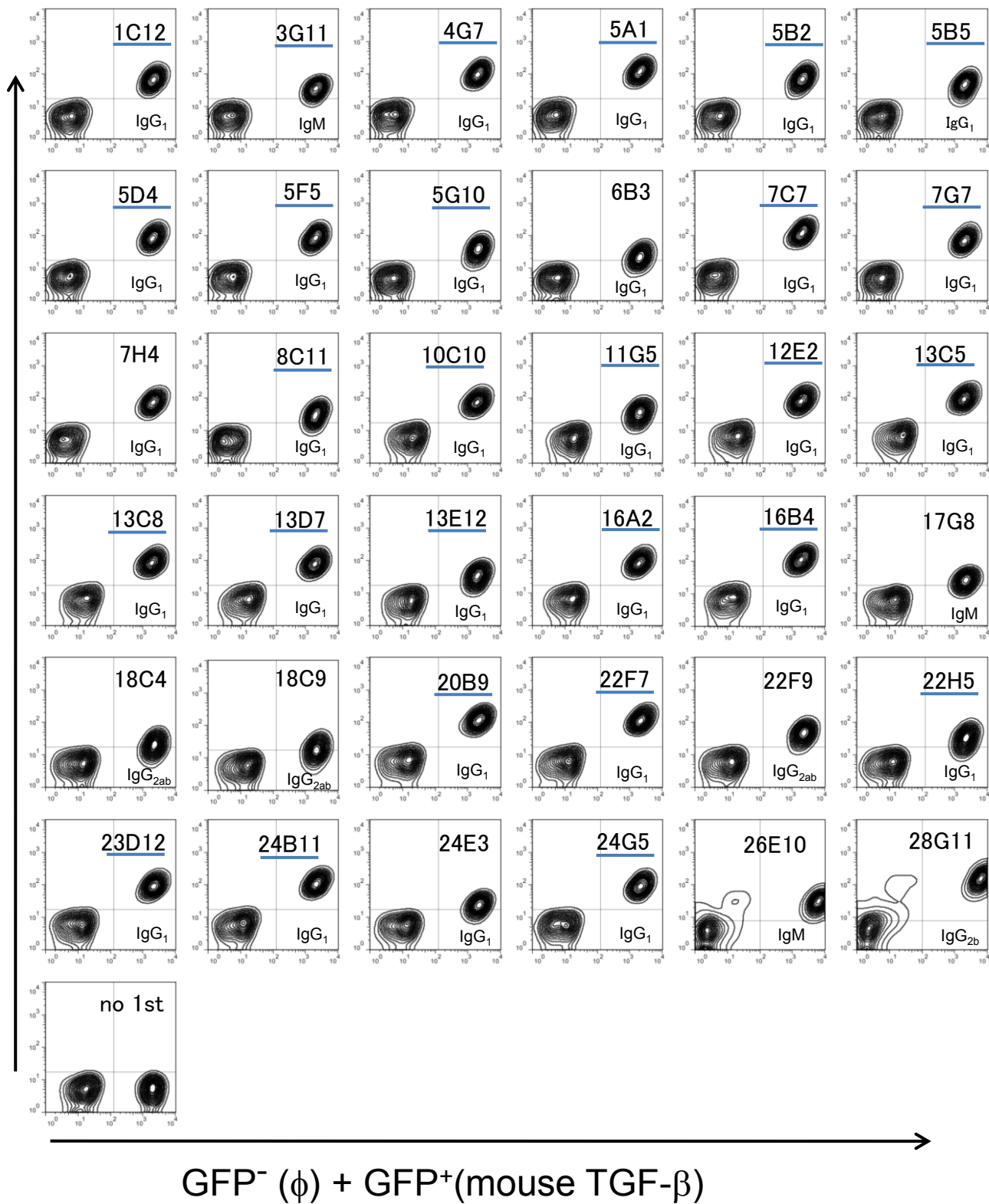

Figure S2

Supplement: Figure S2 — Staining of mouse TGF-β-transduced P3U1 cells with TW7 anti-LAP/TGF-β candidate clones. Mouse TGF-β-transduced P3U1 (clone #11) cells (GFP+) mixed with non-transduced P3U1 cells (GFP(-)) were surface stained with culture supernatants of anti-LAP/TGF-β candidate clones (TW7 series) using goat anti-mouse Ig-APC after Fc receptor blocking. Immunoglobulin subtypes are also shown in the figures. Clones identified as anti-LAP in Fig. 3 are underlined. (PDF) [file pone.0015523.s002.pdf]

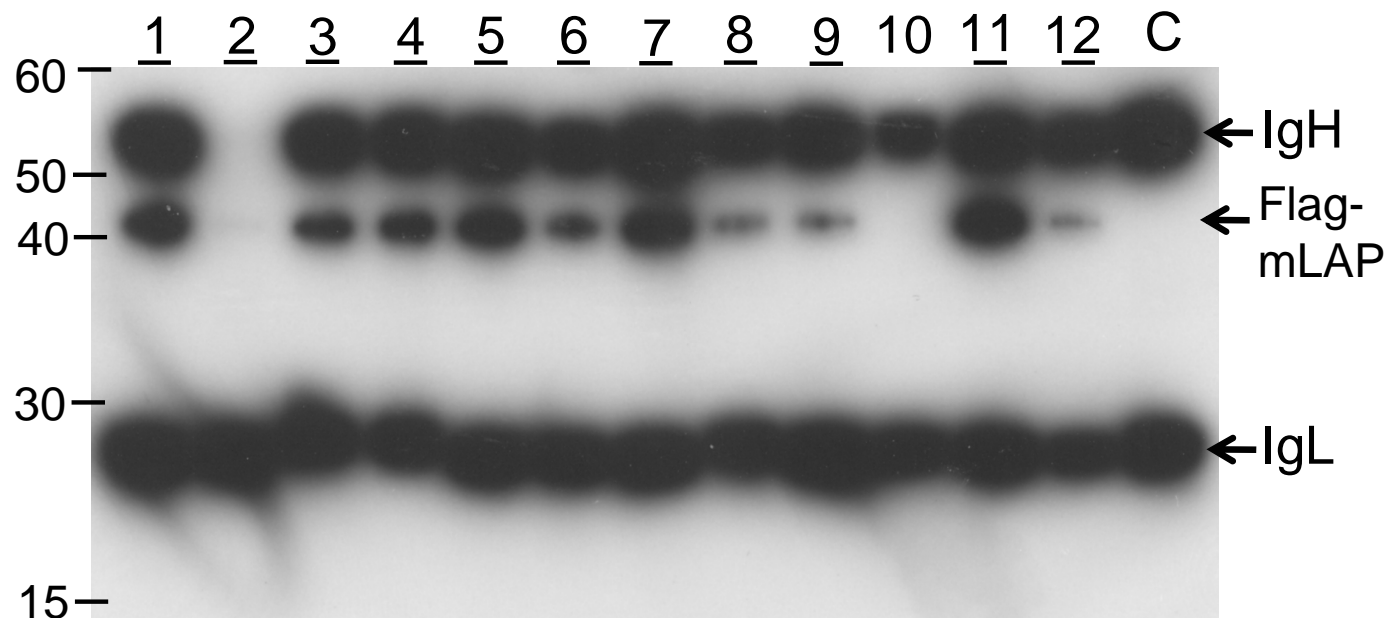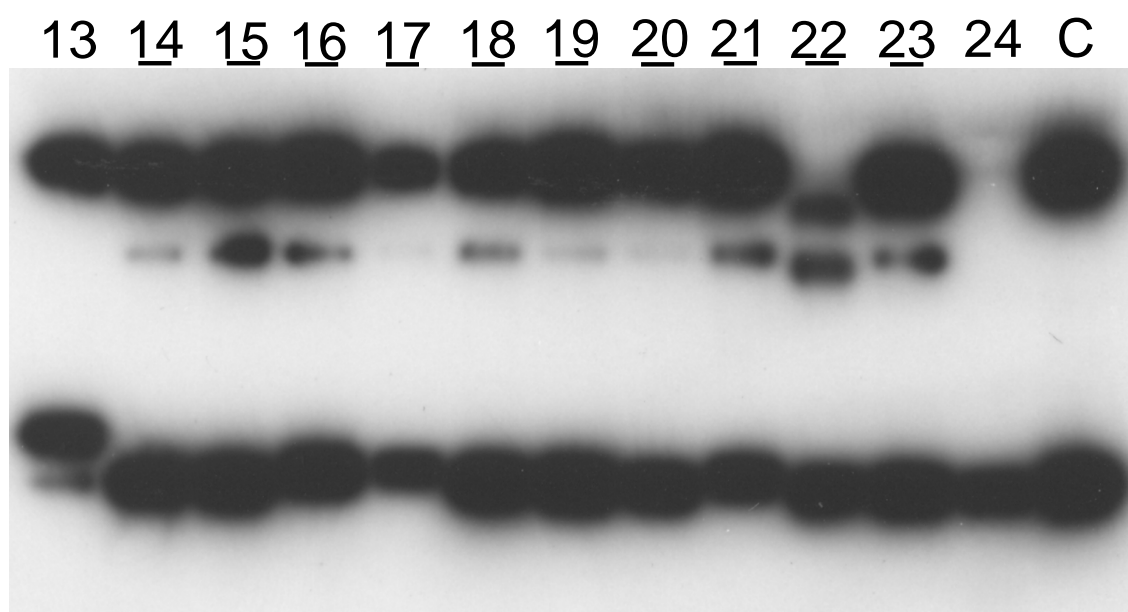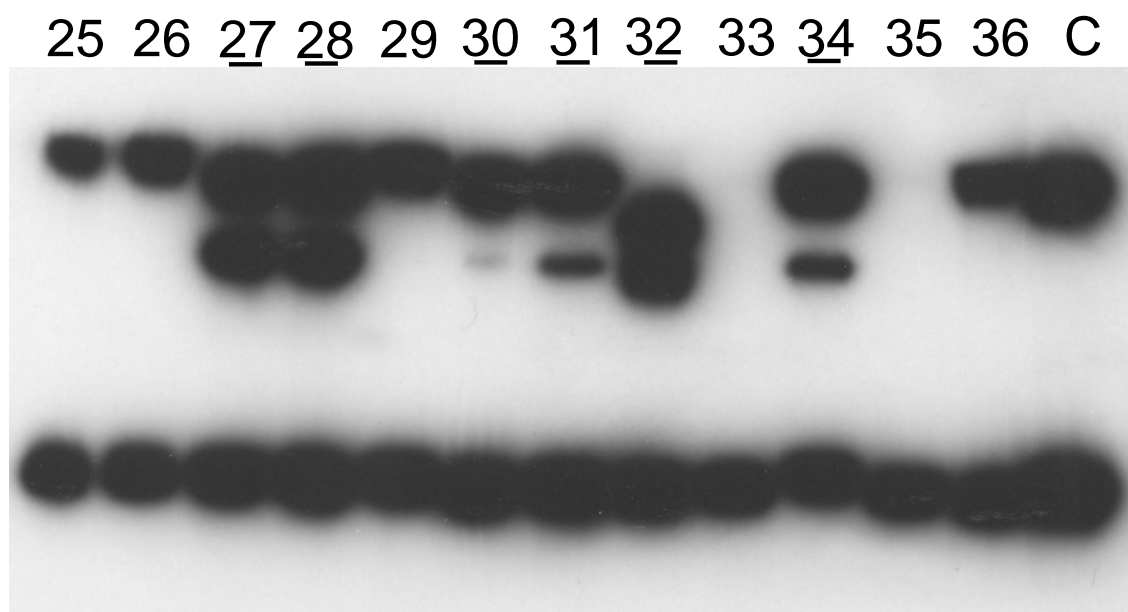

Figure S3

Supplement: Figure S3 — Immunoprecipitation of Flag-tagged mouse LAP with TW7 anti-LAP/TGF-β candidate clones. Culture supernatant of P3U1 cells transduced with retroviral pMCs vector carrying Flag-tagged mouse LAP lacking TGF-β sequence (Flag-mLAP) was immunoprecipitated with anti-LAP/TGF-β candidate clones using anti-mosue IgG BioMag Plus (Polysciences). The immunoprecipitated samples were run on SDS-PAGE under reducing conditions and blotted with anti-Flag mAb M2. Ig H chain and Ig L chain were detected at 55 kDa and at 25 kDa, respectively, and Flag-mLAP migrated at 43 kDa. Clones that immunoprecipitaed Flag-mLAP were marked under the clone numbers. C, MOPC21 IgG1 control; 1, TW7-1C12 (IgG1); 2, TW7-3G11 (IgM); 3, TW7-4G7 (IgG1); 4, TW7-5A1 (IgG1); 5, TW7-5B2 (IgG1); 6, TW7-5B5 (IgG1); 7, TW7-5D4 (IgG1); 8, TW7-5F5 (IgG1); 9, TW7-5G10 (IgG1); 10, TW7-6B3 (IgG1); 11, TW7-7C7 (IgG1); 12, TW7-7G7 (IgG1); 13, TW7-7H4 (IgG1); 14, TW7-8C11 (IgG1); 15, TW7-10C10 (IgG1); 16, TW7-11G5 (IgG1); 17, TW7-12E2 (IgG1); 18, TW7-13C5 (IgG1); 19, TW7-13C8 (IgG1); 20, TW7-13D7 (IgG1); 21, TW7-13E12 (IgG1); 22, TW7-16A2 (IgG1); 23, TW7-16B4 (IgG1); 24, TW7-17G8 (IgM); 25, TW7-18C4 (IgG2a or 2b); 26, TW7-18C9 (IgG2a or 2b); 27, TW7-20B9 (IgG1); 28, TW7-22F7 (IgG1); 29, TW7-22F9 (IgG2a or 2b); 30, TW7-22H5 (IgG1); 31, TW7-23D12 (IgG1); 32, TW7-24B11 (IgG1); 33, TW7-24E3 (IgM); 34, TW7-24G5 (IgG1); 35, TW7-26E10 (IgM); 36, TW7-28G11 (IgG2b). (PDF) [file pone.0015523.s003.pdf]

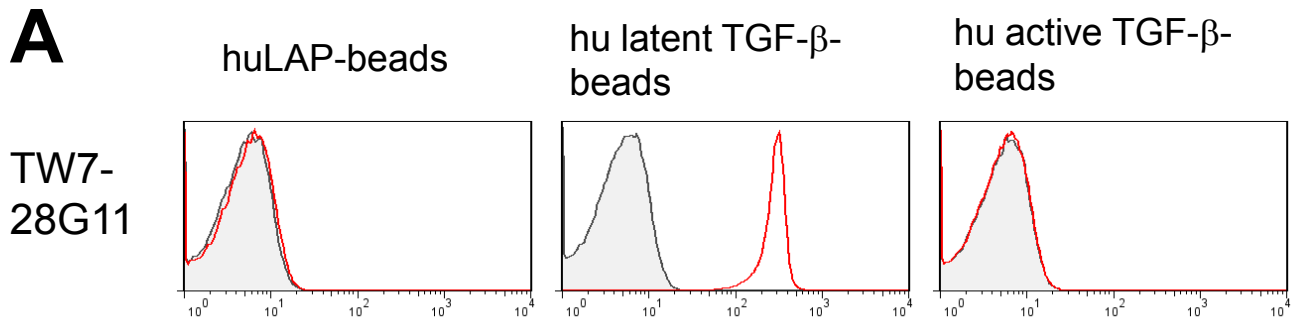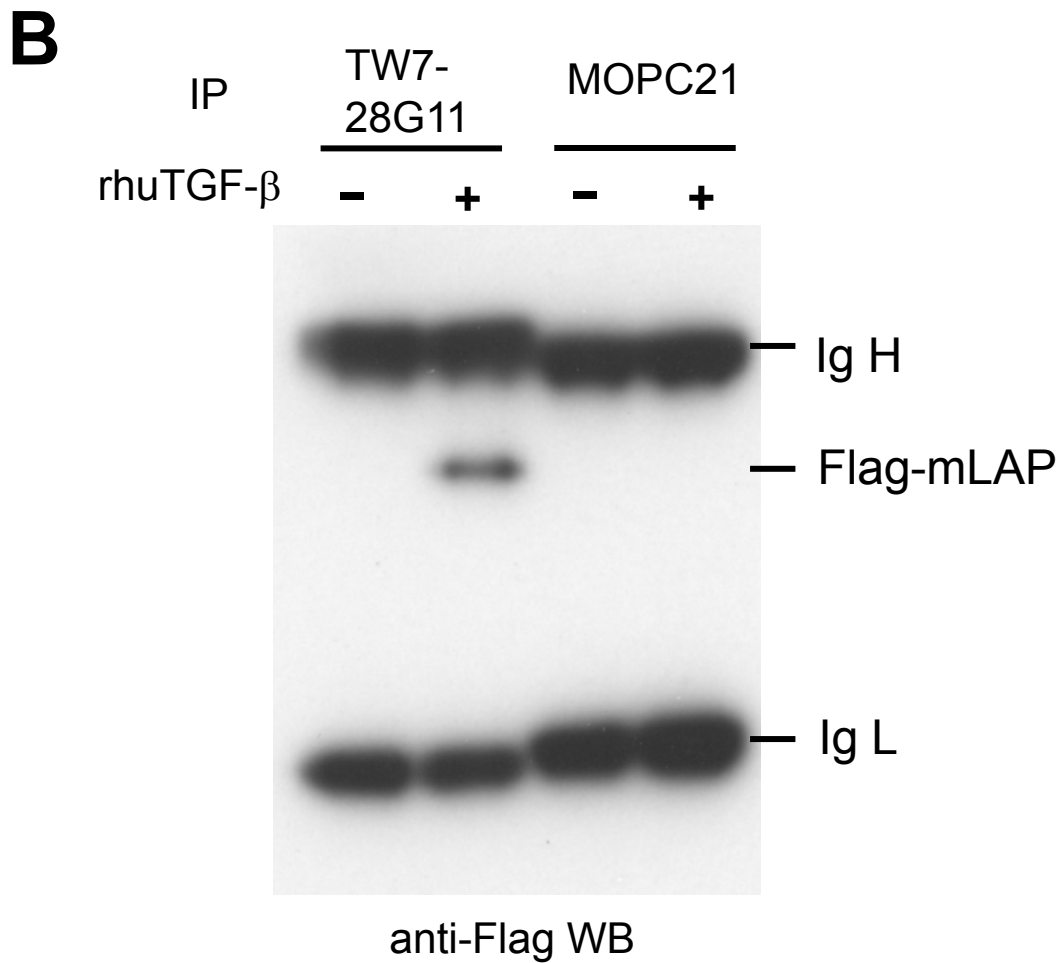

Figure S4

Supplement: Figure S4 — Characterization of TW7-28G11 clone. (A) Recombinant human LAP- (left), human latent TGF-β- (middle), or human active TGF-β- (right) coated polystyrene beads were stained with TW7-28G11 mAb using goat anti-mouse Ig-APC. (B) Culture supernatant of Flag-mLAP-transduced P3U1 cells with/without exogenously added recombinant human TGF-β was immunoprecipitated with TW7-28G11 or control Ab. The samples were run on SDS-PAGE under reducing conditions and blotted with anti-Flag M2 antibody. (PDF) [file pone.0015523.s004.pdf]

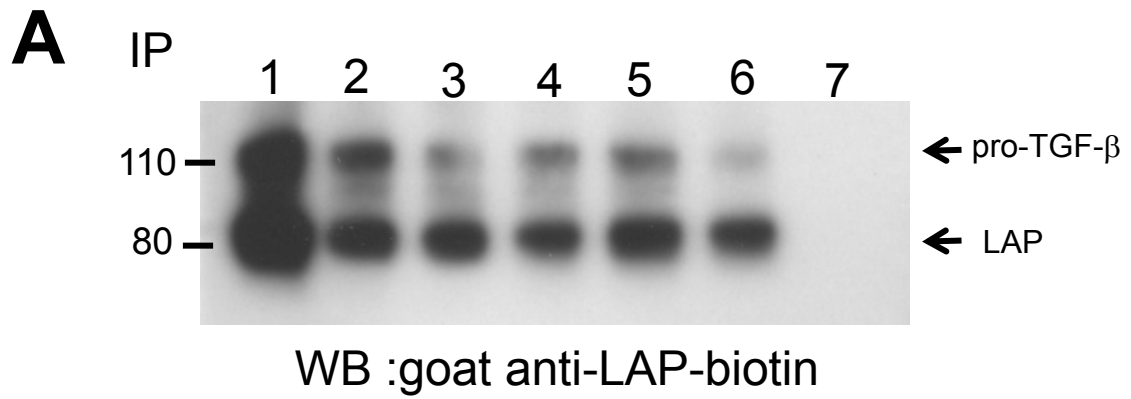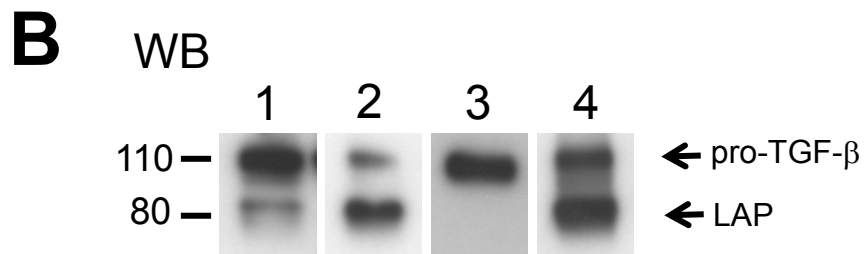

Figure S5

Supplement: Figure S5 — Western blotting and immunoprecipitation of LAP/TGF-β by TW7 mAbs. (A) Culture supernatant of P3U1-muTGF-β (clone #11) cells (lane 1), or immunoprecipitated samples from P3U1-muTGF-β culture supernatant with TW7-7H4 (lane 2), TW7-16B4 (lane 3), TW7-20B9 (lane 4), TW7-22F7 (lane 5), TW7-28G11 (lane 6), or or IgG1 control MOPC21 (lane 7) were run on SDS-PAGE under non-reducing conditions, and blotted with biotinylated goat anti-LAP Ab. (B) Culture supernatant of P3U1-muTGF-β (clone #11) cells were run on SDS-PAGE under non-reducing conditions and blotted with TW7-16B4 (lane 1), TW7-20B9 (lane 2), TW7-28G11 (lane 3), or biotinylated goat anti-LAP (lane 4). (PDF) [file pone.0015523.s005.pdf]

Foxp3-647 (intracellular)

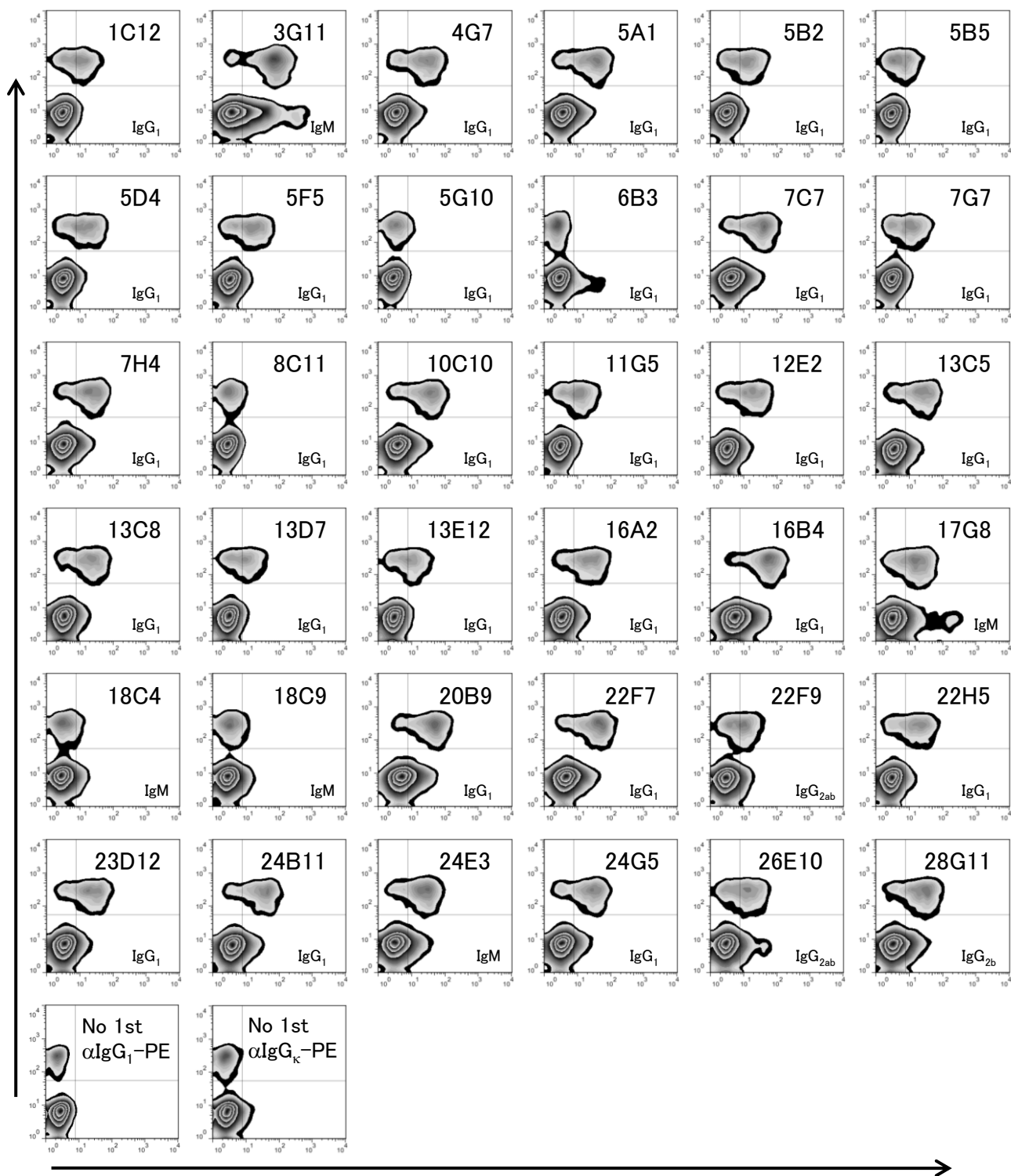

TW7 Ab +  $\alpha\text{IgG}_1\text{-PE}$  or  $\alpha\text{IgG}_{1\kappa}\text{-PE}$  (surface)

Figure S6

Supplement: Figure S6 — Staining of pre-activated mouse CD4 T cells with TW7 anti-LAP/TGF-β mAb series. BALB/c CD4 T cells were stimulated with plate-bound anti-CD3/anti-CD28 for 2 days and rested 1 day. The cells were surface stained with TW7 anti-LAP/TGF-β mAbs using PE-labeled anti-mouse IgG1 or anti-mouse Igκ secondary antibodies, then intracellularly stained with anti-Foxp3-Alexa Fluor647 as Figure 2A. Staining with all 36 TW7 clones was shown. (PDF) [file pone.0015523.s006.pdf]

No  
addition

TGF- $\beta$

ALK5  
inhibitor

$\alpha$ TGF- $\beta$   
1D11

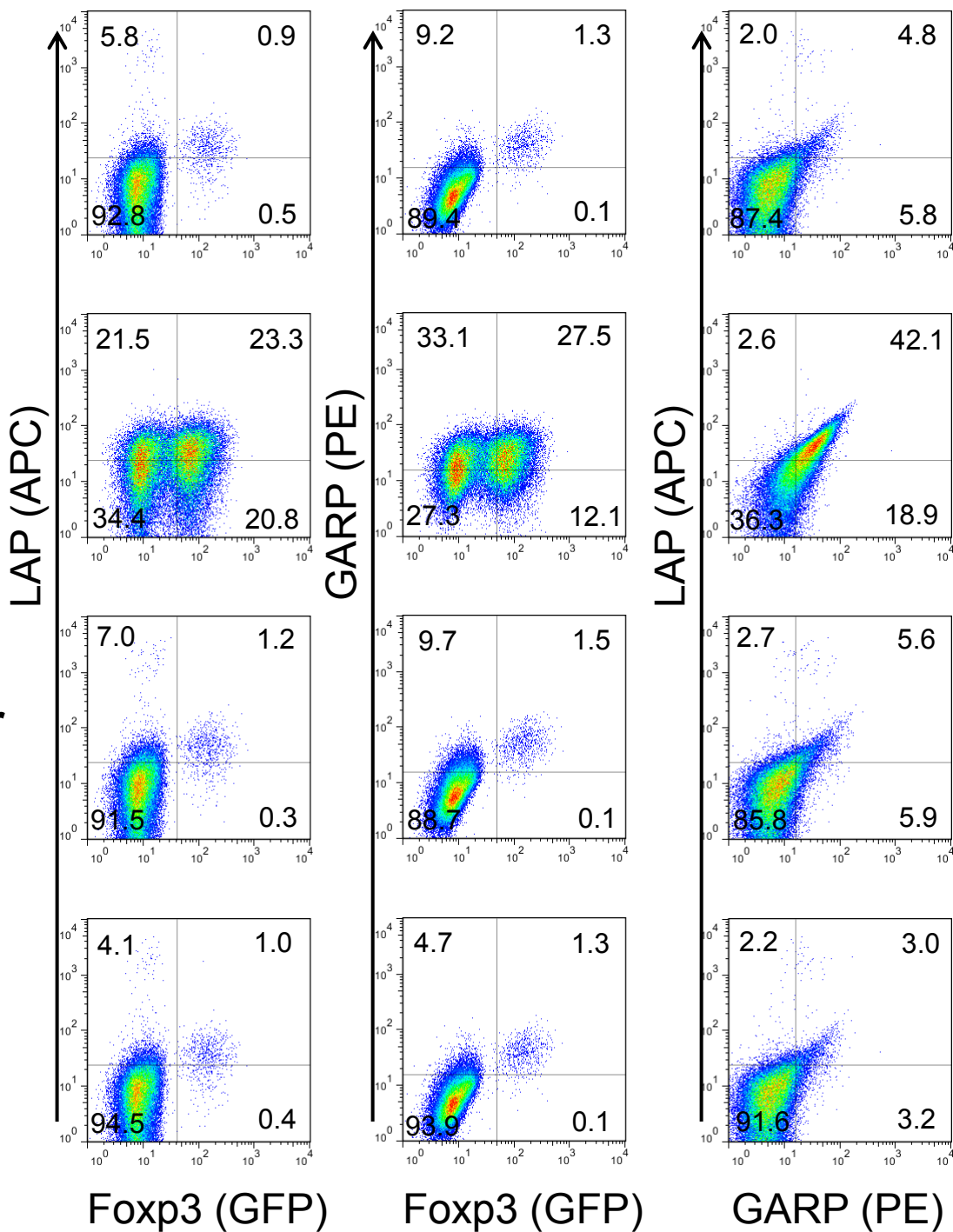

Figure S7

Supplement: Figure S7 — Surface LAP expression under TGF-β blocking conditions. B6 background Foxp3-GFP knock-in CD4 T cells were stimulated with plate-bound anti-CD3/anti-CD28 in presence of 10 ng/ml recombinant human TGF-β, 1 µM ALK5 inhibitor II (Figure S8), or 50 µg/ml anti-TGF-β mAb 1D11 for 2 days, and rested for 1 day. The cells were stained with anti-LAP TW7-16B4 using anti-mouse IgG1-APC secondary antibody and anti-GARP-PE. The quadrants were set by isotype control staining (PDF) [file pone.0015523.s007.pdf]

**A**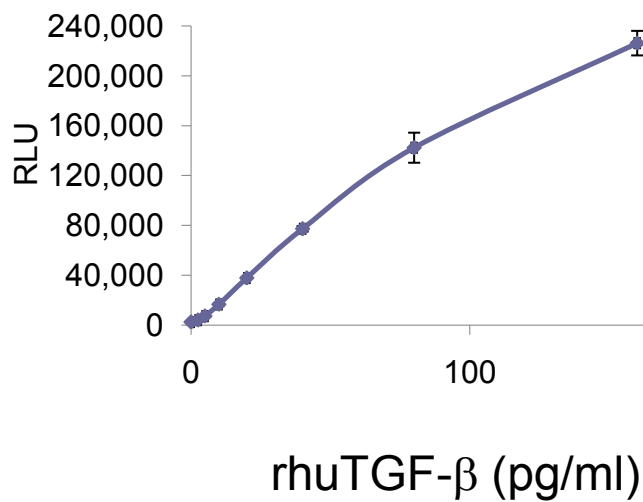**B**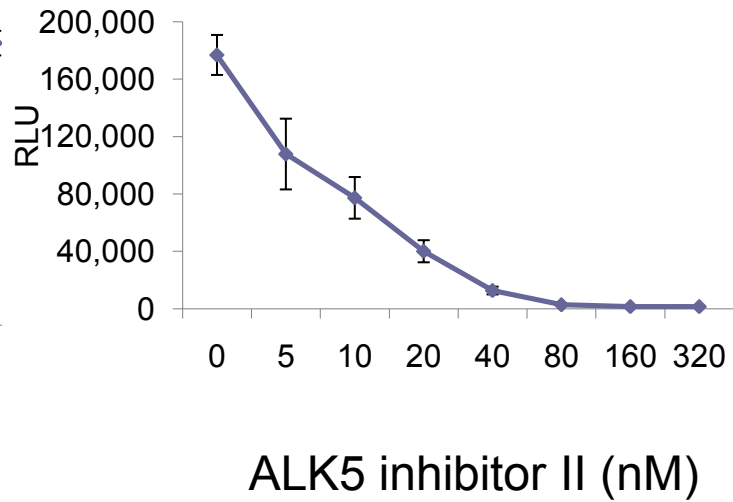

Figure S8

Supplement: Figure S8 — Dose response curve of ALK5 inhibitor II. (A) Mv1Lu cells stably transfected with (caga)12-MLP-Luc vector were cultured in the presence of recombinant human TGF-β for 8 hrs, and luciferase was measured. (B) Mv1Lu-(caga)12-MLP-Luc cells were cultured in presence of 100 pg/ml recombinant human TGF-β with various concentrations of ALK5 inhibitor II for 8 hrs, and luciferease was measured. (PDF) [file pone.0015523.s008.pdf]
